# Two types of ultrafast mode-locking operations from an Er-doped fiber laser based on germanene nanosheets

**DOI:** 10.1007/s12200-023-00068-1

**Published:** 2023-06-07

**Authors:** Baohao Xu, Zhiyuan Jin, Lie Shi, Huanian Zhang, Qi Liu, Peng Qin, Kai Jiang, Jing Wang, Wenjing Tang, Wei Xia

**Affiliations:** 1 School of Physics and Technology, University of Jinan, Jinan, 250022 China; 2 School of Physics and Optoelectronic Engineering, Shandong University of Technology, Zibo, 255049 China; 3 Shandong Huaguang Optoelectronics Co., Ltd., Jinan, 250101 China

**Keywords:** Fiber laser, Germanene, Mode-locked, Noise-like pluses

## Abstract

As a member of Xenes family, germanene has excellent nonlinear saturable absorption characteristics. In this work, we prepared germanene nanosheets by liquid phase exfoliation and measured their saturation intensity as 0.6 GW/cm^2^ with a modulation depth of 8%. Then, conventional solitons with a pulse width of 946 fs and high-energy noise-like pulses with a pulse width of 784 fs were obtained by using germanene nanosheet as a saturable absorber for a mode-locked Erbium-doped fiber laser. The characteristics of the two types of pulses were investigated experimentally. The results reveal that germanene has great potential for modulation devices in ultrafast lasers and can be used as a material for creation of excellent nonlinear optical devices to explore richer applications in ultrafast photonics.

**Graphical Abstract:**

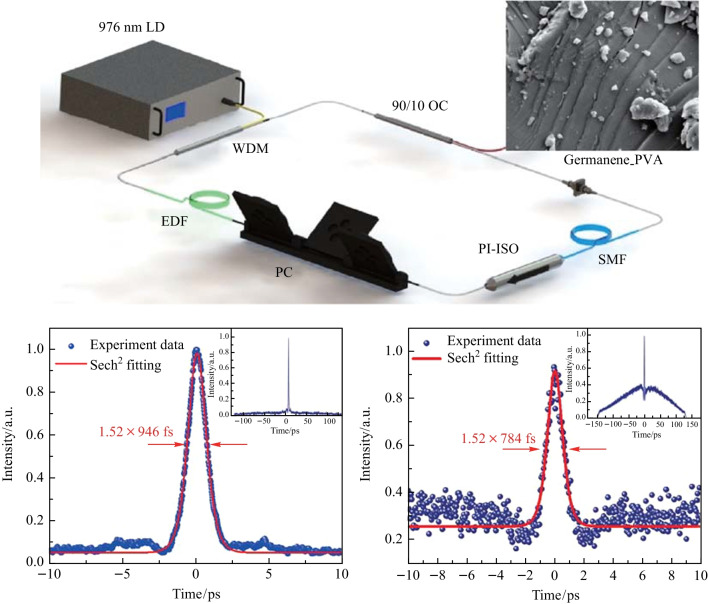

## Introduction

Ultrafast mode-locked fiber lasers with flexible structures have been widely used in various applications due to their high peak power, ultrashort pulse duration, and high stability [1–4]. Among the two common mode-locking techniques, passive mode-locking is superior to active mode-locking due to its easier self-starting, simpler structure, and better environmental stability [5]. In passively mode-locked lasers, saturable absorbers (SAs) are the key devices and can be classified into two types: real SAs and artificial SAs [6, 7]. Although artificial SAs (for example, nonlinear polarization rotation) can enable ultra-fast lasers and high-energy pulses, their high environmental sensitivity and poor stability limit their application. While real SAs with excellent nonlinear optical properties do not have these shortcomings and are being widely investigated by researchers.

In recent years, the discovery of various low-dimensional materials has significantly boosted the development of SAs, which are widely used in mode-locked fiber lasers. From the earliest graphene [8–10], to the later carbon nanotubes (CNTs) [11], transition metal dichalcogenides (TMDs) [12–17], topological insulators (TIs) [18–21], MXenes [22, 23], Xenes [24–26] and other two-dimensional (2D) materials [27, 28], all have been verified to have ultrafast saturable absorption properties. Among them, single-element Xenes, where X denotes possible elements from group IIIA to VIA, and “ene” has Latin origins indicating nanosheets, have been proven to be different from other 2D materials due to their tunable bandgap, ultra-high surface-to-volume ratio, and high carrier mobility [29]. In particular, germanene, which has been studied recently, is proven to have excellent nonlinear optical properties and has aroused widespread research interest in fields of nonlinear optics and ultrafast mode-locked lasers [30, 31]. It has superfast optical response and broadband optical absorption characteristics. In addition, the environmental stability of germanene is good [32]. However, up to now, the research on germanene is still relatively scarce, and its nonlinear optical characteristics in ultrafast optics have not been fully explored.

Based on low-dimensional material SA, passively mode-locked fiber lasers can generate multiple types of solitons. Therefore, passively mode-locked fiber laser is also an ideal device for studying multi-soliton dynamics [33]. By controlling the operating parameters of the laser, such as pump power, polarization state, and cavity length, a variety of soliton states can be obtained, such as soliton rain [34, 35], bound states [36, 37], higher-order harmonics [31], and noise-like pulse states. In particular, compared with the low pulse energy of conventional soliton mode-locking (CS-ML), noise-like mode-locking (NL-ML) can produce larger pulse energy [38]. The high pulse energy of mode-locked pulse in fiber lasers has always been one of the excellent characteristics sought by researchers and NL-ML fiber lasers have great potential for various applications. Usually, most of studies on multi-solitons, especially noise-like pulse (NLP), are realized by using nonlinear polarization rotation or nonlinear amplification ring mirrors, which are artificial SAs. In recent years, investigations on obtaining NL-ML using SAs based on low-dimensional materials have also been gradually reported. For instance, Guo et al. achieved typical NL-ML operation based on WS_2_ in an erbium-doped fiber laser (EDFL), with a spectral bandwidth of 0.48 nm and laser output power of 10.2 mW, corresponding to a single pulse energy of 4.74 nJ [39]. Dong et al. obtained CS-ML with a pulse width of 439 fs and NL-ML operation with a pulse width of 1.75 ps based on single-wall CNTs at 1550 nm [40]. Zhao et al. used PbS quantum-dots as the SA in an EDFL to obtain NL-ML operation with a pulse duration of 1.6 ps and a pulse energy of 9.68 nJ [41]. However, investigations of the NL-ML operation based on real SAs are still insufficient. Therefore, it is very important to explore novel 2D SAs for mode-locking fiber lasers and investigate corresponding mechanisms.

In this paper, we have successfully prepared the germanene-PVA thin film and investigated its nonlinear optical characteristics. Then, the germanene-PVA SA was applied in an EDFL. CS-ML operation with a minimum pulse duration of 946 fs and a signal-to-noise ratio (SNR) of 80 dB was obtained at low pump powers, corresponding to a maximum pulse energy of 0.13 nJ. When the single pulse energy exceeded the energy range of the CS-ML pulse as the pump power increased, NL-ML operation was achieved with a pulse energy of 0.4 nJ, a SNR of 75 dB, and a coherent peak half-height width of 784 fs. This work indicates that germanene can be used as an excellent saturable absorber material for achieving high-energy mode-locked pulses.

## Preparation and characterization of SA

**Fig. 1:**
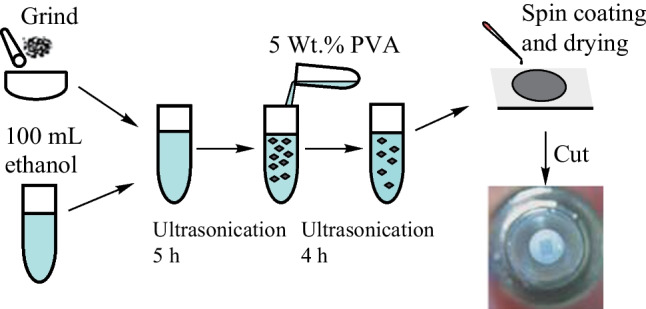
Preparation of germanene-PVA thin films

Germanene nanosheets were prepared by the method of liquid phase exfoliation (LPE). The preparation steps are shown in Fig. 1. First, the bulk germanium powder was ground into fine particles in a grinding dish, and subsequently, the ground germanium powder was added to a container containing 100 mL ethanol. Then the mixture was ultrasonically shaken for 5 h with an ultrasonic cleaner and centrifuged at 1500 r/min for 20 min. Next, the supernatant was taken and mixed with 5 Wt.% PVA solution in a 1:1 ratio. After 4 h of ultrasonic treatment, a uniformly distributed germanene-PVA dispersion was obtained. Finally, the germanene-PVA solution was dropped onto a clean flat glass substrate and then placed in an oven at 35 °C for 4 h to obtain the germanene-PVA film. A 1 mm × 1 mm size germanene-PVA film was cut out and connected with a flange and sandwiched between two fiber end faces as SA.

**Fig. 2:**
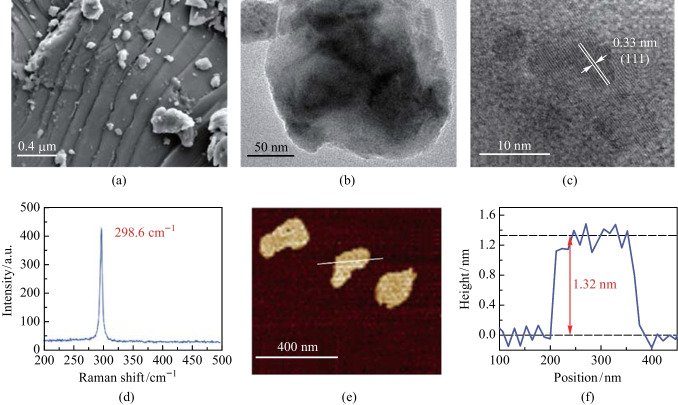
**a** SEM image of germanene, **b** TEM image, **c** HRTEM image, **d** Raman spectrum, **e** AFM image, and **f** the corresponding thicknesses curve of the germanene nanosheets

To investigate the morphology and optical characteristics of germanene nanosheets, several characterization methods were carried out. First, as shown in Fig. 2a, a scanning electron microscopy (SEM, ZEISS Sigma 300) image of the ground germanene powder was obtained. Since the interlayer force of germanene is a weak van der Waals force, a distinct layered structure can be seen in the SEM image. The layered structure of the nanosheets can be seen by transmission electron microscopy (TEM, JEOLJEM 2100) and is shown in Fig. 2b. Figure 2c shows the high-resolution TEM (HRTEM, JEOLJEM 2100) images of germanene nanosheets, from which the lattice spacing of 0.33 nm could be determined. The Raman spectra (Horiba LabRAM HR Evolution) presented in Fig. 2d show that there is a typical Raman peak of germanene nanosheets at 298.6 cm^−1^, which corresponds to the in-plane vibration mode (*E*_2g_). The Atomic Force Microscope (AFM, Bruker Dimension Icon) was used to measure the thickness of germanene nanosheets, and the AFM image is given in Fig. 2e. Figure 2f illustrates the corresponding thickness curve. It was found that the thickness of the germanene nanosheet was 1.32 nm, corresponding to about 4 layers of germanene [42].

**Fig. 3:**
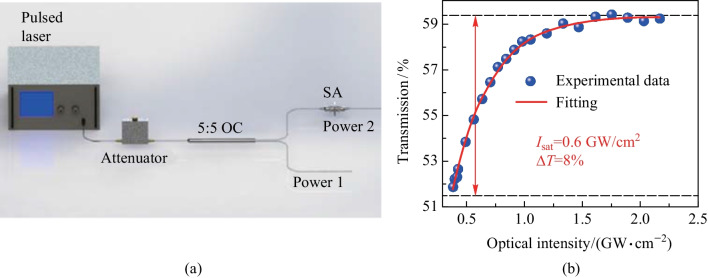
**a** Measurement setup diagram of the dual-power detection method, **b** transmission curve of the germanene-PVA SA

Subsequently, the nonlinear optical characteristics of germanene nanosheets were investigated by the dual-power detection method. Figure 3a shows the measurement setup diagram. A 1550 nm ultra-short pulse laser was used as the light source with a repetition frequency of 12.93 MHz and a pulse duration of 1.1 ps. The transmittances at different output powers are shown in Fig. 3b. The experimental data were fitted by the formula $T\left(I\right)=1-{T}_{\mathrm{ns}}-\Delta T\cdot \mathrm{exp}(-I/{I}_{\mathrm{sat}})$, in which $T\left(I\right)$ and $I$ are the transmission and the input intensity, respectively. The fitting value of modulation depth $\Delta T$ was 8%, corresponding to a saturation intensity *I*_sat_ of 0.6 GW/cm^2^. The unsaturated loss *T*_ns_ was about 40.6%.

## Experimental setup

**Fig. 4:**
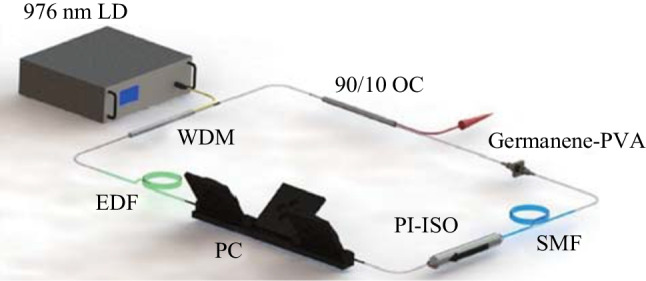
Diagram of the experimental setup of the Germanene-PVA film passively mode-locked fiber laser

The experimental setup of the Germanene-PVA film passively mode-locked fiber laser is schematically illustrated in Fig. 4. A 976 nm laser diode (LD) was adopted as the pump light source with a maximum output power of 600 mW. The gain medium was a 0.3 m long erbium-doped fiber (EDF), whose group velocity dispersion (GVD) was 22.95 ps^2^/km. The intracavity polarization state was regulated by the polarization controller (PC). The device before the PC was a polarization-independent isolator (PI-ISO) to ensure unidirectional light propagation in the laser resonant cavity. A 90:10 output coupler (OC) was used to output the 1550 nm lasing light. A thin film of germanene-PVA with a size of 1 mm × 1 mm was embedded directly between two fiber connectors as the SA. A 6.2 m long single-mode fiber (SMF) was added to make the laser operate in the negative dispersion region and reduce the repetition rate. The total cavity length was 6.5 m. The group dispersion coefficient of SMF was − 21.68 ps^2^/km. The net dispersion of the cavity was − 0.128 ps^2^. The pulse properties were measured by a 500 MHz mixed oscilloscope (Wavesurfer 3054z) combined with a high-speed photo-detector (PD-03), an optical spectrum analyzer (Anritsu MS9710C), a commercial autocorrelator (FR-103XL), an RF spectrum analyzer (Agilent N9020A), and an optical power meter.

## Results and discussion

At first, to confirm that the mode-locking pulse was generated by modulation of the germanene-PVA SA, we removed the germanene nanosheet, and no matter how we adjusted the PC and pump input power (*P*_in_) no mode-locked pulse was generated, indicating that the germanene nanosheet played a critical role in the generation of the mode-locking pulse.

**Fig. 5:**
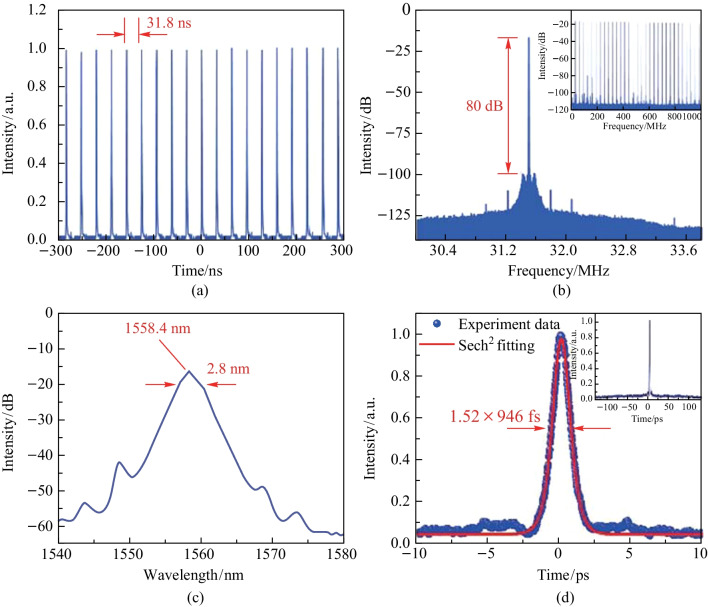
Characteristics of the CS-ML pulses at the* P*_in_ of 251 mW: **a** the pulse trace, **b** the RF spectrum, **c** the optical spectrum, and **d** the autocorrelation trace with sech^2^ fitting curve

Adding the SA into the cavity, stable CS-ML pulses could be obtained by carefully tuning the PC. Here, a phenomenon of *P*_in_ hysteresis appeared. Increasing* P*_in_ to 111.8 mW, CS-ML pulses could be first found. Then, reducing *P*_in_ to 60.4 mW, the CS-ML could still operate stably. However, if *P*_in_ continued to decrease until the CS-ML pulse disappeared, the pulse could only reappear when *P*_in_ increased back to the threshold power of 111.8 mW. The trace of the mode-locked pulse sequence was recorded by oscilloscope at *P*_in_ of 251 mW and illustrated in Fig. 5a. The time interval between adjacent pulses was 31.8 ns, which corresponded to a repetition frequency of 31.49 MHz. As shown in Fig. 5b, a significant SNR of 80 dB was measured. The RF spectrum in the range of 1 GHz is illustrated in the inset of Fig. 5b, showing a very consistent and stable peak, proving that the CS-ML pulse had excellent stability. Figure 5c shows the optical spectrum of the CS-ML pulse. The spectral central wavelength of the CS-ML pulse was 1558.4 nm, with a 3 dB bandwidth of 2.8 nm. The Kelly sideband caused by the dispersive wave indicated that the laser was operating in the negative dispersion region. The Kelly sidebands look a bit blurry due to the strong absorption of the resonant continuous wave background signal caused by the zero-bandgap structure of germanene SA [43]. Finally, under the *P*_in_ of 251 mW, the pulse width of the CS-ML pulse was recorded by the autocorrelation instrument. The full width half-maximum (FWHM) of the autocorrelation trajectory was 1.46 ps, as shown in Fig. 5d. After fitting, the pulse width of 946 fs could be obtained. The corresponding time-bandwidth product (TBP) was 0.328, which was marginally larger than the theoretical value of the hyperbolic secant pulse of 0.315, indicating the existence of a minor amount of chirp.

**Fig. 6:**
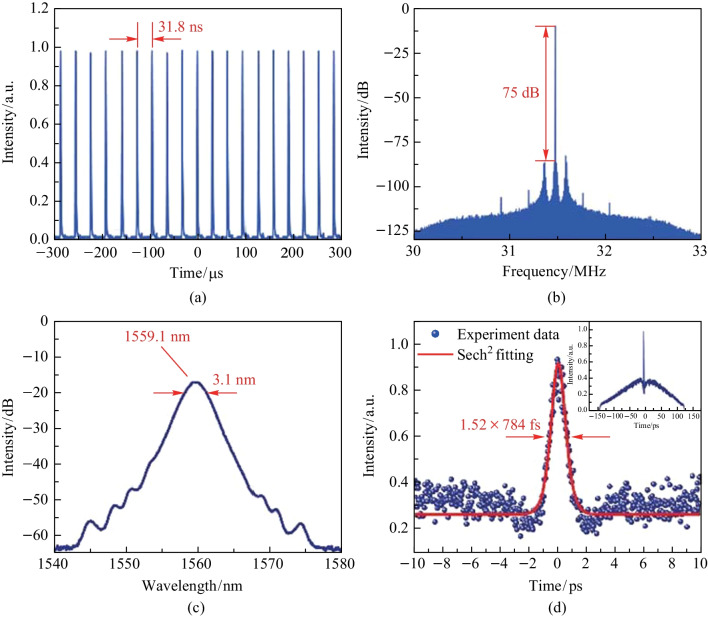
Characteristics of the NLP at the *P*_in_ of 261.1 mW: **a** the pulse trace, **b** the RF spectrum, **c** the optical spectrum, and **d** the autocorrelation trace with sech^2^ fitting curve

As the *P*_in_ continued to increase, typical NL-ML pulses could be obtained in the same laser cavity when the Pin reached 261.1 mW. This state occurred mainly caused by the interaction of multiple solitons. When the *P*_in_ increased further, the CS-ML state became destabilized, and the soliton collapsed. The characteristics of NL-ML pulses are shown in Fig. 6. Figure 6a gives the pulse trajectory diagram at the *P*_in_ of 261.1 mW. The NL-ML pulse operated at the basic repetition frequency, so it had the same pulse interval (31.8 ns) and repetition frequency (31.49 MHz) as the CS-ML pulse. The SNR of 75 dB was obtained and is shown in Fig. 6b, indicating that the NL-ML pulses had the same high stability as CS-ML pulses. Figure 6c demonstrates the optical spectrum of the NL-ML pulse with the spectral center wavelength at 1559.1 nm. The corresponding 3 dB bandwidth was 3.1 nm, which was larger than that of the CS-ML pulse. The autocorrelation trajectory diagram in Fig. 6d is quite different from that of the CS-ML pulse, showing a narrow coherent peak on a wide base, this is a typical characteristic of NL-ML pulse [44]. The NL-ML pulse was equivalent to a pulse envelope composed of many ultrafast sub-pulses with diverse pulse duration and peak powers. The FWHM of the coherent spike was 1.21 ps, corresponding to the pulse width of 784 fs. The TBP of 3.30 could be calculated by the pulse width and spectral bandwidth.

**Fig. 7:**
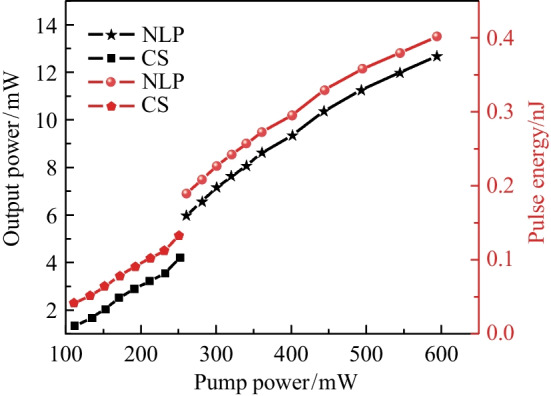
Average output powers and single pulse energies of CS-ML pulse and NL-ML pulse against *P*_in_

The output powers and single-pulse energies of both CS-ML pulses and NL-ML pulses versus the *P*_in_ were recorded and are given in Fig. 7. Stable CS-ML pulse was achieved in the *P*_in_ range of 111.8 to 251.6 mW, and noise-like operation was achieved in the range of 261.1 to 595.7 mW. At *P*_in_ of 251.6 mW, the maximum output power of the CS-ML pulse was 4.23 mW, and the single pulse energy at this time was 0.13 nJ, which was consistent with the energy of conventional solitons (~ 0.1 nJ). For the NL-ML pulse, the maximum output power was 12.71 mW at *P*_in_ of 595.7 mW, corresponding to a single pulse energy of 0.4 nJ, which was much higher than that of the CS-ML pulse.

**Fig. 8:**
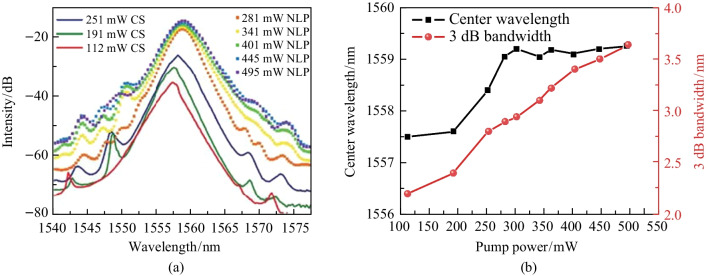
**a** Spectra at different *P*_in_. **b** curves of spectral bandwidth and center wavelength with different *P*_in_

To further investigate the spectral changes under different pulse mechanisms, we recorded the spectra for different *P*_in_ values, as shown in Fig. 8. Compared with the CS-ML pulse, the spectrum of the NL-ML pulse was much smoother with a wider spectral width. However, because the single pulse energy was not large enough, there were still small sidebands in the noise-like spectra, and we believe that the spectra could have been wider as well as smoother if the *P*_in_ had continually increased. We also measured the variation of the spectral center wavelength and 3 dB bandwidth versus the *P*_in_. Whether in CS-ML or NL-ML operation, the 3 dB bandwidth of the laser increased with the increase of the* P*_in_. When the *P*_in_ was less than 251 mW, the laser operated in the CS-ML state and the center wavelength increased with the increase of *P*_in_; when the *P*_in_ was greater than 261 mW, the laser entered the NL-ML state and the center wavelength was almost unchanged.

Finally, to verify the long-term stability of the fiber laser, the oscilloscope traces and optical spectra of CS-ML and NL-ML pulses were all observed for three continuous periods of 4 h, once every day for three days, at the pump powers of 251 and 597 mW. The pulse train and the central wavelength of the spectrum were always stable. No damage on the PVA film was found. It was thus experimentally demonstrated that the EDFL with germanene nanosheets as the SA can produce highly stable CS-ML pulses and large energy NL-ML pulses. It was also shown that germanene material has great potential to be used in SA devices for the generating of large energy ultrashort pulses.

## Conclusion

In summary, CS-ML pulse and NL-ML pulse were successfully obtained in an EDFL using a germanene-PVA SA prepared by liquid phase stripping method. Both states were operated at a repetition frequency of 31.49 MHz. The spectral bandwidth of conventional solitons was 2.8 nm, corresponding to the pulse width of 946 fs and the single pulse energy of 0.13 nJ at a pump input power of 251 mW. In the noise-like state, the pulse width of NL-ML pulses was 784 fs, with the maximum single pulse energy of 0.4 nJ. The research confirms that the germanene material can play a great role in the study of nonlinear dynamics.

## Data Availability

The data that support the findings of this study are available from the corresponding author, upon reasonable request.
